# Enhanced Antibacterial Performance and Cytocompatibility of Silver Nanoparticles Stabilized by Cellulose Nanocrystal Grafted with Chito-Oligosaccharides

**DOI:** 10.3390/ma11081339

**Published:** 2018-08-02

**Authors:** Xiaohui Ni, Jinru Wang, Yiying Yue, Wanli Cheng, Dong Wang, Guangping Han

**Affiliations:** 1 Key Laboratory of Bio-based Material Science and Technology (Ministry of Education), Northeast Forestry University, Harbin 150040, China; hnixiaohui@hotmail.com (X.N.); wangjinrunefu@outlook.com (J.W.); nefucwl@nefu.edu.cn (W.C.); zcwangd@hotmail.com (D.W.); 2 College of Biology and the Environment, Nanjing Forestry University, Nanjing 210037, China; yue@njfu.edu.cn

**Keywords:** carboxy-cellulose nanocrystals, chito-oligosaccharides, silver nanoparticles, antibacterial activity, cytocompatibility

## Abstract

The agglomeration of silver nanoparticles (AgNPs) results in poor antibacterial performance, and the accumulation of silver in the human body threatens human health. Preparing a matrix is a technique worth considering as it not only prevents the aggregation of AgNPs but also reduces deposition of AgNPs in the human body. In this paper, carboxy-cellulose nanocrystals (CCNC) were prepared by a simple one-step acid hydrolysis method. Chito-oligosaccharides (CSos) were grafted onto the surface of CCNC to form CSos-CCNC composite nanoparticles. CCNC and CSos-CCNC were used as stabilizers for deposing AgNPs and two types of complexes—AgNPs-CCNC and AgNPs-CSos-CCNC—were obtained, respectively. The influence of the two stabilizer matrices—CCNC and CSos-CCNC—on the morphology, thermal behavior, crystal structure, antibacterial activity, and cell compatibility of AgNPs-CCNC and AgNPs-CSos-CCNC were examined. The results showed that the AgNPs deposited on the CSos-CCNC surface had a smaller average diameter and a narrower particle size distribution compared with the ones deposited on CCNC. The thermal stability of AgNPs-CSos-CCNC was better than that of AgNPs-CCNC. AgNPs did not affect the crystalline structure of CCNC and CSos-CCNC. The antibacterial activity of AgNPs-CSos-CCNC was better than that of AgNPs-CCNC based on antibacterial studies using *Escherichia coli*, *Staphylococcus aureus*, and *Klebsiella pneumoniae.* The cytotoxicity of AgNPs-CSos-CCNC was remarkably lower than that of AgNPs-CCNC.

## 1. Introduction

In recent years, silver nanoparticles (AgNPs) have been extensively investigated for their appealing properties and applied to many fields, such as bacteriostat [1,2], conductive adhesives [3,4], metal-enhanced fluorescence, surface-enhanced Raman scattering [5,6], catalytic fuel degradation [7,8], etc. Due to their low toxicity, high thermal stability, and low volatility, they may also be used in water purification, food preservation, cosmetics, and so on [9]. As a well-known broad-spectrum antibacterial agent [10], AgNPs are accessible and cost-effective compared with most current antibiotics. While several studies have shown the ease with which certain bacteria evolve resistance to silver, most bacteria will not become drug-resistant to AgNPs due to their unique antibacterial mechanism [11,12]. However, AgNPs, which are small and unstable, will aggregate into large particles, resulting in reduced antimicrobial performance. Therefore, dispersing AgNPs uniformly in the composite material is important for realizing their desired function.

Fabrication of nanocomposites on the nanoscale is a simple method to develop or modify novel structural or functional heterogeneous material [13]. AgNPs are useful materials, and incorporating a chemically robust nanoscale matrix is a promising method to fabricate functional AgNP complexes. It is sustainable to select a suitable matrix and use renewable or naturally derived nanomatrix to fabricate bio- or green-nanocomposites. At present, one prevalent trend is to utilize cellulose nanocrystals (CNC) as a green matrix to combine functional groups or nanoparticles to achieve desired purposes. Biocompatible and biodegradable CNC can be extracted from higher plants or certain bacteria through many controlled methods, such as physics, chemistry, and biology. The driving force to explore CNC as matrix in functional nanocomplexes is the fact that their nanodegree diameter and length contribute to large specific areas and enable easy passage through the cell membrane. Moreover, multitude amounts of reactive groups of -OH in the structure, as well as extremely high crystallinity, enable high mechanical strength and thermal stability. Accordingly, CNC are one of the most promising nanomatrices to process functional nanocomposites. Examples of incorporating CNC into functional nanocomposites include biotemplate material, enzyme immobilization, heterogeneous catalysis, biosensor, bioimaging, and drug carriers [14]. CNC have been demonstrated to serve as matrix material in obtaining inorganic nanoparticles with particle size that are characteristically monodisperse, nonagglomerated, and narrowly distributed in size. However, the distinct contribution of CNC to controllable design and further improvements in the tailor-made microstructure and performances of the functional nanocomposites remain limited.

There have been many studies on the synthetic method [15], the antimicrobial mechanism [16], and antimicrobial performance [8] of AgNPs. However, there is little literature about reducing the deposition of AgNPs in the body. Peng et al. [17] first compared the cytotoxicity of AgNPs stabilized by poly(vinylpyrrolidone), chito-oligosaccharides (CSos), and pure AgNPs. The result indicated that AgNPs stabilized by chito-oligosaccharides could significantly reduce the deposition amount on the liver of mice in seven days. The mechanism of chito-oligosaccharides reducing the deposition amounts of AgNPs in mice body may be attributed to the huge amounts of amino groups in chito-oligosaccharides since protonated amino can adsorb many materials with low molecular weight, such as metal ions, cholesterol, triglycerides, cholic acid, and organic mercury. Because chito-oligosaccharides originate from the chitin that is widely found in nature, chito-oligosaccharides are easily dissolved and have lower cytotoxicity compared with chitosan. Therefore, chito-oligosaccharides are also ideal green AgNP stabilizers.

In this study, carboxy-cellulose nanocrystals (CCNC) were prepared by a one-step acid hydrolysis method. CCNC are the products of partial oxidation of hydroxyl groups on the surface of CNC to carboxyl groups. Chito-oligosaccharides were grafted onto the surface of CCNC by peptide coupling reaction, and they were used as the stabilizer to fabricate AgNPs. CCNC were also used as the stabilizer to fabricate AgNPs. The main objective of this study was to investigate the influence of CCNC and CSos-CCNC on the surface morphology and properties of AgNPs. The effects of the two stabilizers on the physico-chemical properties, antibacterial properties, and cytocompatibility of AgNPs were explored to provide a theoretical basis for the preparation of AgNPs with high antibacterial activity and low deposition.

## 2. Materials and Methods

### 2.1. Materials

α-Cellulose (diameter = 90 μm), ammonium persulfate [(NH_4_)_2_S_2_O_8_, APS], 1-ethyl-3-(3-dimethylaminopropyl)-carbodiimide (EDC, AR grade), *N*-Hydroxy succinimide (NHS, >98%), 2-(*N*-morpholino) ethane sulfonic acid (MES, >99%), silver nitrate (AgNO_3_, 99.9%), NaBH_4_, nutrient broth powder, Nutrient Agar, Plate Count Agar, fetal bovine serum, dual anti-streptomycin, glutamine, DMEM 1640 basal medium, rhodamine were purchased from the Aladdin Chemistry Co., Ltd. (Shanghai, China). *Escherichia coli* (*E. coli*), *Klebsiella pneumoniae*, and *Staphylococcus aureus* (*S. aureus*) bacteria were purchased from Guangdong Institute of Microbiology (Guangdong, China). Amoxicillin powder was purchased from Fuyao Pharmaceuticals Co., Ltd. (Fuzhou, China). The human pancreatic cancer cell lines PANC-1 were obtained from ATCC, and the normal cell lines NCTC clone 929 (L929) were purchased from Gaining Biological Co., Ltd. (Shanghai, China). CCK-8 cell proliferation and cytotoxicity detection kit were purchased from Beyotime Institute of Biotechnology. Deionized water was used throughout the study. All chemicals were used as received without any further purification.

### 2.2. Preparation of CCNC

Three grams of α-cellulose was added to 100 mL of 2 M APS solution. The mixture was vigorously stirred at 62 °C for 24 h. The reacted suspension was washed four times with deionized water by centrifugation (8000 rpm, 10 min) to remove the inorganic ion until the solution pH was neutral. The water of the resulting suspension was removed by freeze-drying, and white powder was obtained.

### 2.3. Preparation of CSos-CCNC

The process of grafting CSos onto CCNC was performed according to Bulpitt P [3] with some modifications. 0.2 g CCNC and 0.2 g EDC were dispersed in deionized water (100 mL) and sonicated for 15 min. A 25 mL solution containing NHS (0.11 g) was subsequently added to the suspension and sonicated for 5 min. Then, a 75 mL solution used to dissolve CSos (0.36 g) was added to the mixture. The pH of the solution was adjusted to 5 by adding 2 M NaOH, and 1.95 g MES were used as buffer to keep the pH of the solution constant. The mixture was vigorously stirred for 24 h ambient temperature. After the reaction, the solution was dialyzed against deionized water for at least 48 h to remove free molecules from suspension, and the suspension was then freeze-dried to obtain light yellow powder.

### 2.4. Preparation of AgNPs Stabilized by Nanoparticles

Following a typical procedure of preparing AgNPs stabilized with CCNC (AgNPs-CCNC), 425 mg of AgNO_3_ was introduced into 250 mL of deionized water to prepare the aqueous solution of AgNO_3_. Then, 15 mL of AgNO_3_ solution was added into a flask containing 30 mL of 1 mg/mL CCNC suspension. The flask was incubated in ice-water bath. Fifteen milliliters of freshly prepared 0.01 M NaBH_4_ was added to the suspension drop by drop under stirring until the color of the mixture solution changed to black. The mixture solution was continuously stirred for 1 h and then washed with deionized water four times by centrifugation (8000 rpm, 10 min). Finally, the AgNPs-CCNC powder was collected by lyophilizing in a vacuum freeze dryer. The procedure of preparing AgNPs stabilized with CSos-CCNC (AgNPs-CSos-CCNC) was the same as the procedure of preparing AgNPs-CCNC.

### 2.5. Characterization

The morphology of AgNPs-CCNC and AgNPs-CSos-CCNC were observed by transmission electron microscopy (TEM, JEM-2100, JEOL, Tokyo, Japan) at an accelerating voltage of 200 kv. The samples of AgNPs-CCNC were prepared by dropping the AgNPs-CCNC suspension (1 mg/mL) onto a 400 mesh carbon-coated copper grid. The sample was stained with a droplet of phosphotungstic acid to enhance image contrast. For samples observed only for AgNPs, there was no procedure of phosphotungstic acid staining. The particle dimensions were calculated from the TEM images using the nanomeasurer software (Fu Dan University, Shanghai, China). For each sample, 100 particles were randomly selected and measured.

Fourier transform infrared (FTIR) spectroscopy analysis were performed using a Nicolet 50 (Thermo Fisher scientific, Waltham, MA, USA) to detect various chemical bonds. FTIR spectral were recorded from 4000 to 500 cm^−1^ with a resolution of 4 cm^−1^ and an accumulation of 64 scans. The samples were mixed with dried potassium bromide (KBr) powder (1% sample in anhydrous KBr) and pressed into a pellet. The degree of oxidation (DO) of CCNC was obtained by calculating the ratio of the peak height near 1730 cm^−1^ and the one near 1050 cm^−1^, corresponding to the absorption of carbonyl and cellulose backbone, respectively.

Thermal gravimetric analysis (TGA) was obtained using STA 6000-SQ8 (Perkinelmer company, Waltham, MA, USA) by heating the samples from 40 °C to 800 °C at a heating rate of 10 °C/min in a nitrogen atmosphere with a flow rate of 20 mL/min. The wide-angle X-ray diffraction (XRD) analysis was carried out on a X, Pert3 Powder X-ray diffractometer (Panalytical B.V company, Almelo, Netherlands) by using Cu Kα (1.5418 Å) X-ray. Diffraction data were collected from 5° to 80° at the rate of 5°/min at ambient temperature.

The antibacterial activity of the resultant AgNPs-CSos-CCNC and AgNPs-CCNC nanoparticles were evaluated using Gram-negative (*E. coli* and *Klebsiella pneumoniae*) and Gram-positive (*S. aureus*) bacteria by determining the minimum inhibitory concentration (MIC) [7]. The technique of using MIC was in accordance with a previous method described in literature, with minor modifications. First, freshly prepared bacterial suspensions were diluted to an absorbance value of 0.1–0.2 at 600 nm (equivalent to 0.5 McFarland standards). Next, 200 µL of diluted bacterial suspension was mixed with 2 mL liquid medium containing a certain quantity of samples in a sterilized 10 mL test tube. Then, the suspensions were placed onto a rotary shaker at 100 rpm and kept at 37 °C for 3 h. Afterwards, 150 µL of the supernatant was transferred to the surface of an agar plate and spread evenly to cover the surface using a sterile glass rod in a sterile environment. Finally, the agar plates were incubated at 37 °C overnight and the MIC of AgNPs-CSos-CCN and AgNPs-CCN were determined.

The cytotoxicity of the resultant AgNPs-CSos-CCNC and AgNPs-CCNC were evaluated by CCK8. Typically, pancreatic ductal cancer cells (Panc-1) and mouse fibroblast cells (L929) were seeded in 96-well plates (3 × 10^3^ cells per well) filled with 100 µL of cell medium and incubated at 37 °C in a humidified atmosphere of 5% CO_2_ for 24 h. Then, 10 µL of freshly prepared cell mediums containing different concentrations of AgNPs-CSos-CCNC and AgNPs-CCNC were used to replace the counterpart volume of culture medium and incubated for another 24 h. Ten concentrations were examined: 2.00, 1.75, 1.50, 1.25, 1.00, 0.75, 0.50, 0.25, 0.10, and 0 mg/mL. Finally, 10 µL CCK8 solution was added to each well and incubated at 37 °C for 1 h. The absorbance was measured at 450 nm using a photometric microplate reader. The average readings and standard deviations were based on four samples, and all tests were performed in triplicate. The cell viability values were calculated according to the following equation: Cell viability (%) = (the absorbance of experimental group/the absorbance of control group) × 100%.

## 3. Results and Discussion

### 3.1. The Morphology and Structure of AgNPs-CCNC

**Figure 1:**
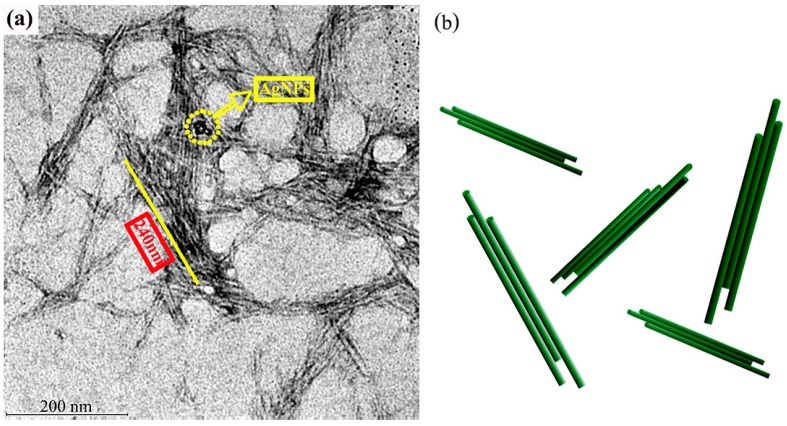
(**a**) TEM image of silver nanoparticles-carboxy-cellulose nanocrystals (AgNPs-CCNC) and (**b**) schematic illustrations of cellulose bundles.

The microstructure of AgNPs-CCNC was observed by TEM (Figure 1). APS hydrolysis removed the amorphous regions of the cellulose fibers but left behind the crystalline regions intact, resulting in short aggregated CCNC bundles, as shown in Figure 1a. Schematic illustrations of CCNC bundles are shown in Figure 1b.

Most of the rod-shaped cellulose nanocrystals were aggregated in bundles; this can be attributed to the hydrogen bond attraction between different cellulose nanocrystals due to unoxidized hydroxyl groups on the surface of CCNC. Individual CCNC bundles with negligible sidewise and longitudinal connection union were also clearly identified, ensuring a prerequisite condition for using CCNC as the nanocarrier. From TEM images, the average diameter and length of the CCNC bundles was 22 ± 7 nm and 240 ± 40 nm, respectively, indicating that the corresponding aspect ratio was ~11. The widespread black spots were AgNPs.

### 3.2. Characterizations of CCNC and CSos-CCNC

**Figure 2:**
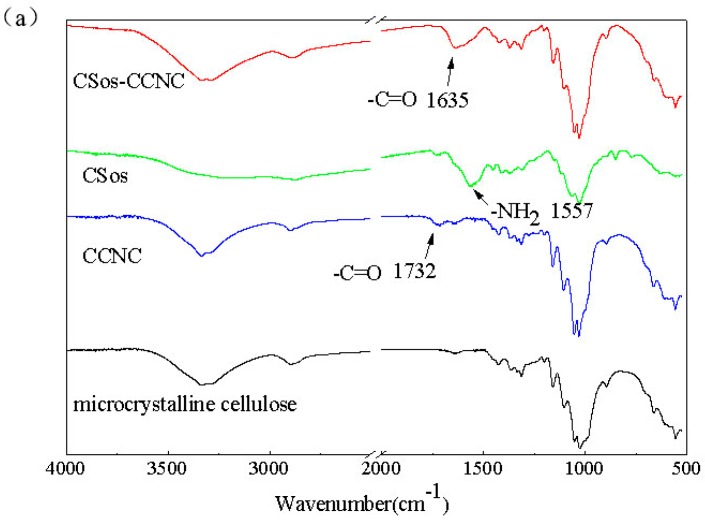

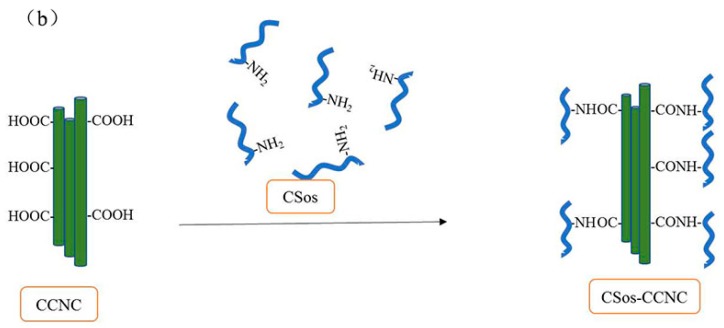
(**a**) FTIR spectrums of microcrystalline cellulose (MCC), CCNC, chito-oligosaccharides (CSos), and CSos-CCNC and (**b**) schematic of the synthesis process of CSos-CCNC.

The typical FTIR spectra of microcrystalline cellulose (MCC), CCNC, CSos, and CSos-CCNC are shown in Figure 2a, and the mechanism diagram of the synthesis process for CSos-CCNC is presented in Figure 2b.

As shown in Figure 2a, the characteristic infrared bands of MCC were observed at the band between 3600 and 3000 cm^−1^ (O-H stretching vibration), the band between 3000 and 2800 cm^−1^, 1500 cm^−1^, and 1250 cm^−1^ (aliphatic C-H stretching vibrations and bending vibrations of CH_2_). The peaks in the finger print region at 1160 and 1070 cm^−1^ (bending and asymmetrical stretching vibration of C-O-C in the glucose) are associated with the saccharide structure. It is notable that the spectrum for the CCNC prepared in APS medium was similar to that of MCC except for a new peak shown by the arrow at 1732 cm^−1^ (C=O carbonyl stretching), which validated the obtaining of CCNC [18,19]. As previously reported [19,20], the spectrum of CSos is similar to that of cellulose as their chemical structures are similar apart from the bonds at 1557 cm^−1^ (-NH_2_ bending stretching) and 1732 cm^−1^ (C=O carbonyl stretching). CSos-CCNC was obtained by esterification of the -OH on the surface of CCNC with NH_2_ on the surface of CSos, as depicted by the mechanism diagram (Figure 2b). As expected, the spectrum of CSos-CCNC was identical to those of CCNC and CSos and displayed characteristic bands of both materials. However, some changes were observed in the grafted composite. For CSos-CCNC, a new peak was observed at 1635 cm^−1^, which validated the formation of new amide bond between CCNC and CSos. In the formation process of the new amine bond, the carbonyl bond was shifted to a lower frequency and buried under the water peak, and the -NH_2_ bending vibration was shifted to a higher frequency coinciding with the carbonyl stretching vibration in amides.

To the best of our knowledge, there are two strategies to determine the degree of oxidation (DO) value for CCNC. One strategy is conducted by conductivity titration, while the other is based on the peak height ratio of a particular group in the infrared spectrum. The result of the conductivity titration was in agreement with that obtained by the FTIR spectrum [21]. In the latter method, the DO value could be obtained by calculating the ratio between carbonyl peak intensity and the most powerful bond near 1070 cm^−1^ related to the skeleton of cellulose. The DO value could also be found by using the formula: DO = 0.01 + 0.7 (I1732/I1060) [22,23], where I indicates the intensity of the absorption band near 1730 cm^−1^. Here, the DO value of CCNC obtained from infrared spectra is 0.076. Theoretically, the maximum DO value of CCNC is 0.2 [21]. It is deemed that around 38% of primary hydroxyl was oxidized to -COOH. The 62% -OH residue contributed to the agglomeration of CCNC. In addition, there was no peak at 1732 cm^−1^ in the spectra of CSos-CCNC, which indicated that most of the carboxyl groups were consumed.

### 3.3. The Morphology of AgNPs

**Figure 3:**
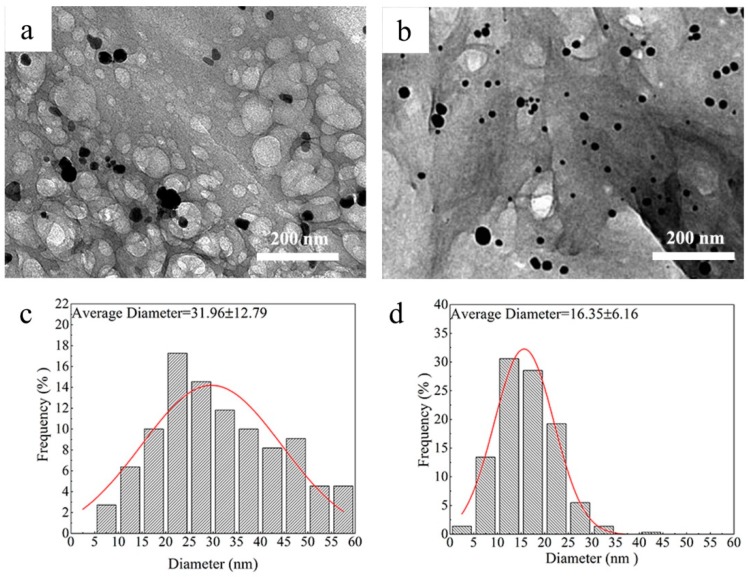
TEM images and size diameter distribution of AgNPs stabilized on (**a**,**c**) CCNC and (**b**,**d**) CSos-CCNC.

Further proof that AgNPs were stabilized by either CCNC or CSos-CCNC was revealed by TEM. The image and the corresponding size histograms are shown in Figure 3.

AgNPs were prepared by the reduction of AgNO_3_ with NaBH_4_ in an ice-water bath using CCNC and CSos-CCNC as the stabilizer. It could be clearly found that the AgNPs deposited on both stabilizers showed homogeneous spherical-shaped and well-dispersed morphology (Figure 3a,b), which indicated that both CCNC and CSos-CCNC were good stabilizers for AgNPs. Notably, as observed from Figure 3c,d, the average diameter of AgNPs stabilized on the CCNC matrix was noticeably larger than those stabilized with CSos-CCNC. The improved dispersibility of CSos-CCNC on AgNPs might be related to the fact that some AgNPs were deposited on CSos instead of CCNC, which meant that the grafted CSos enlarged the specific surface area of CCNC and contributed to more small-sized AgNPs compared to that deposited only on CCNC.

### 3.4. Thermal Property of AgNPs-CCNC and AgNPs-CSos-CCNC

**Figure 4:**
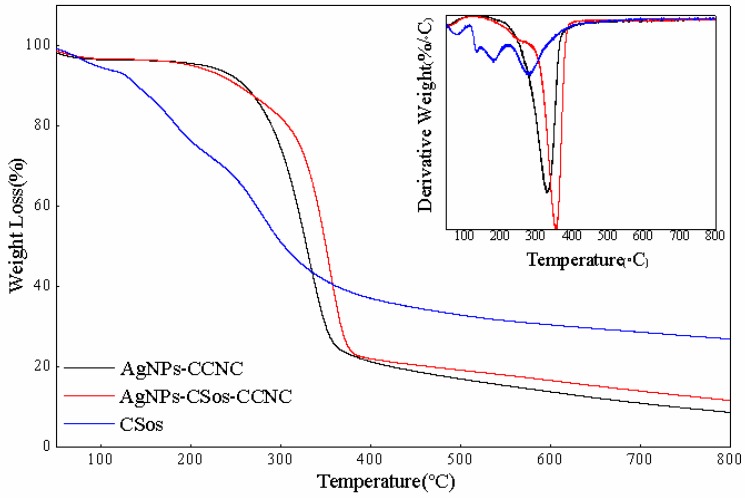
Thermal gravimetric analysis (TGA) curves of AgNPs-CCNC and AgNPs-CSos-CCNC; the inserts correspond to differential thermogravimetry (DTG) curves of samples.

TGA was used to investigate the effect of the two stabilizer matrices on the thermal stability of AgNPs-CCNC and AgNPs-CSos-CCNC complexes, and the results are displayed in Figure 4 and Table 1.

Two stages of weight loss were observed for these two complexes. The small decline that occurred below 100 °C was prompted by the water evaporation retained in the materials. The next weight loss stage observed at temperature ranging from 200 °C to 400 °C could be induced by the thermal decomposition of the substrate materials. Both composites had only one decomposition peak, illustrating the compatibility between CCNC, CSos, and AgNPs and further indicating that AgNPs were uniformly dispersed in the matrix [18].

In the degradation stage, as shown in Figure 4 and Table 1, the T_onset_ of AgNPs-CCNC was higher than that of AgNPs-CSos-CCNC. This phenomenon could be ascribed to the degradation of CSos in AgNPs-CSos-CCNC, as CSos were decomposed from room temperature and had a wide decomposition interval. Furthermore, the T_max_ of AgNPs-CCNC and AgNPs-CSos-CCNC were 330.18 °C and 353.41 °C, respectively, indicating that the thermal stability of AgNPs-CSos-CCNC was superior to that of AgNPs-CCNC, which might have been triggered by the stable chemical link between CSos and CCNC. In addition, the residual mass of AgNPs-CCNC and AgNPs-CSos-CCNC nanoparticles were 8.15% and 11.19%, respectively. The 3.04 wt.% weight loss difference between AgNPs-CSos-CCNC and AgNPs-CCNC nanoparticles also confirmed the presence of CSos in the AgNPs-CSos-CCNC nanoparticles. Finally, as AgNPs were not decomposed under experimental conditions, the amount of AgNPs in AgNPs-CCNC was less than 11%, and the counterpart in AgNPs-CSos-CCNC was under 8%.

### 3.5. Structural Characteristics of the AgNPs-CCNC and AgNPs-CSos-CCNC

**Figure 5:**
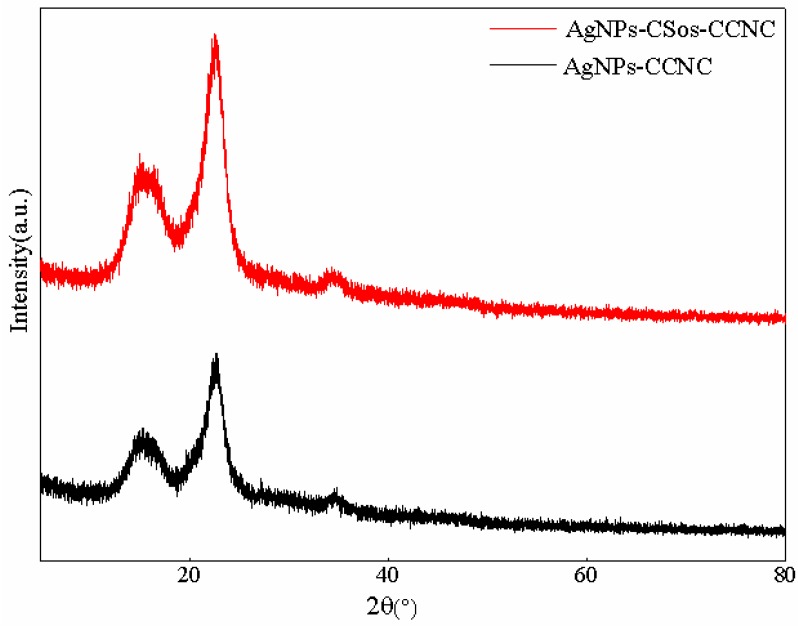
XRD curves of AgNPs-CCNC and AgNPs-CSos-CCNC.

XRD analysis was used to investigate the influence of AgNPs on the structural characteristics of CCNC and CSos-CCNC complexes, and the results are presented in Figure 5.

The structural stability of CCNC and CSos-CCNC is an important factor for its application. However, the structural difference between AgNPs, CCNC, and CSos-CCNC and the changing microstructure of the obtained complexes made it difficult to simply predict the structural characteristics of AgNPs-CCNC and AgNPs-CSos-CCNC. Therefore, XRD analysis of AgNPs-CCNC and AgNPs-CSos-CCNC were conducted. It could be observed that there were no obvious differences between the two complexes. There were three prominent peaks at 2θ = 15.2°, 22.9°for both XRD profiles, which were indexed as a plane of cellulose crystalline (110) and (200) [24]. Interestingly, there was no peak indexed as AgNPs. The absence of diffraction peaks on AgNPs can be explained by a combination of the small quantity of AgNPs and diffraction widening of AgNPs.

### 3.6. Antimicrobial Property of AgNPs-CCNC and AgNPs-CSos-CCNC

As a very effective antimicrobial medicament, AgNPs have been widely used in many commodities. Although the sterilization mechanism is not very clear [25,26], it is certain that the feature of stable dispersion is one indispensable reason behind its superior performance. Here, we compared the influence of AgNPs deposited on CCNC and CSos-CCNC on the antimicrobial activity against *E. coli*, *S. aureus*, and *Klebsiella pneumoniae.*

**Figure 6:**
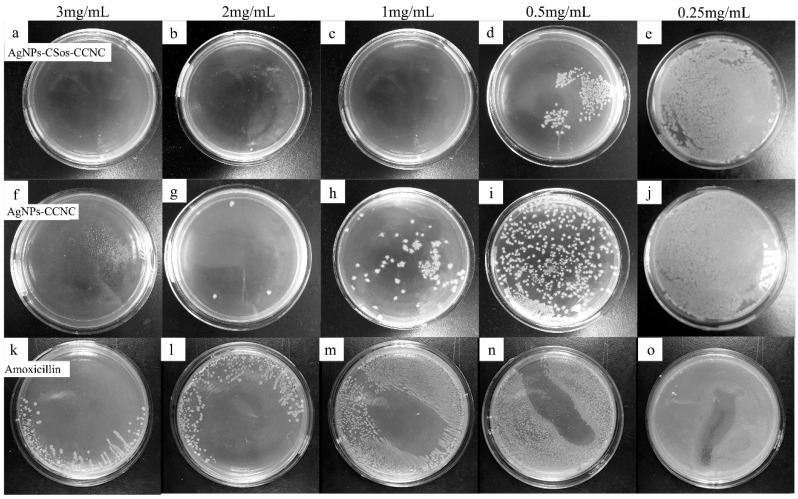
Antimicrobial activity of AgNPs-CSos-CCNC, AgNPs-CCNC and amoxicillin evaluated with *E. coli*. The antimicrobial agent concentrations were shown on the top of each plate. (**a**–**e**) the colony growth with AgNPs-CSos-CCNC, (**f**–**j**) the colony growth with AgNPs-CCNC, (**k**–**o**) the colony growth with amoxicillin.

The result of the MIC test for *E. coli* is shown in Figure 6. The antimicrobial agent concentrations are shown on the top of each plate. The plates in the top row show the community growth with AgNPs-CSos-CCNC, while the plates in the middle row show the community growth under AgNPs-CCNC. The plates in the bottom row show the community growth under amoxicillin as positive control.

The bacterial community density decreased gradually with increased amounts of AgNPs. The *E. coli* was completely wiped out when the concentration of AgNPs-CCNC reached 3 mg/mL (Figure 6f), while the AgNPs-CSos-CCNC complexes against *E. coli* required concentration of 1 mg/mL (Figure 6c). The MIC for AgNPs-CSos-CCNC was between 0.5 and 1 mg/mL compared to 2–3 mg/mL for AgNPs-CCNC. The *E. coli* colonies were still observed when the concentration of amoxicillin was 3 mg/mL. Therefore, the antimicrobial activity of AgNPs-CSos-CCNC was about three times better than that of AgNPs-CCNC.

**Figure 7:**
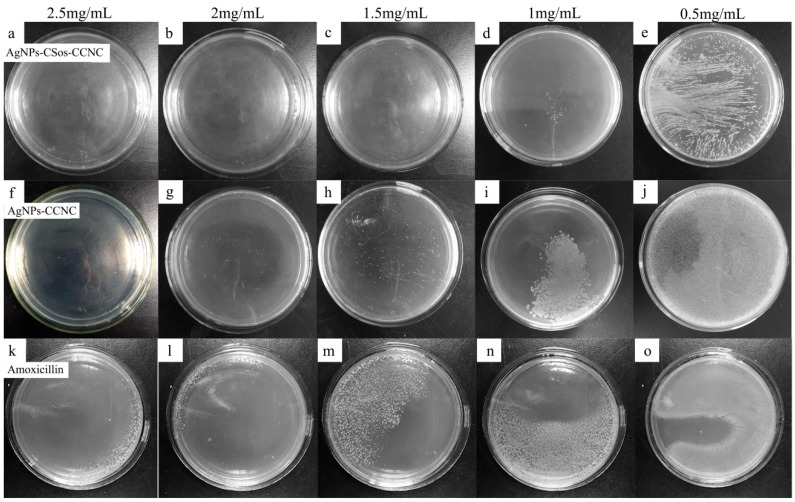
Antimicrobial activity of AgNPs-CSos-CCNC, AgNPs-CCNC and amoxicillin evaluated with *S. aureus.* The antimicrobial agent concentrations were shown on the top of each plate. (**a**–**e**) the colony growth with AgNPs-CSos-CCNC, (**f**–**j**) the colony growth with AgNPs-CCNC, (**k**–**o**) the colony growth with amoxicillin.

The result of MIC test for the *S. aureus* system is shown in Figure 7. The antimicrobial agent concentrations are shown on the top of each plate. The plates in the top row show the community growth with AgNPs-CSos-CCNC, while the plates in the middle row show the community growth under AgNPs-CCNC. The plates in the bottom row show the community growth under amoxicillin as positive control.

The bacterial community density decreased with increasing amounts of AgNPs. The *S. aureus* were entirely extinguished when 2.5 mg/mL AgNPs-CCNC was used (Figure 7f), while for AgNPs-CSos-CCNC, the same effect acquired 1.5 mg/mL of AgNPs-CCNC (Figure 7c). The MIC for AgNPs-CSos-CCNC was between 1 and 1.5 mg/mL compared to 2–2.5 mg/mL for AgNPs-CCNC. The *S. aureus* colonies were observed when the concentration of amoxicillin was 2.5 mg/mL. Therefore, the antimicrobial activity of AgNPs-CSos-CCNC was about two times better than that of AgNPs-CCNC.

**Figure 8:**
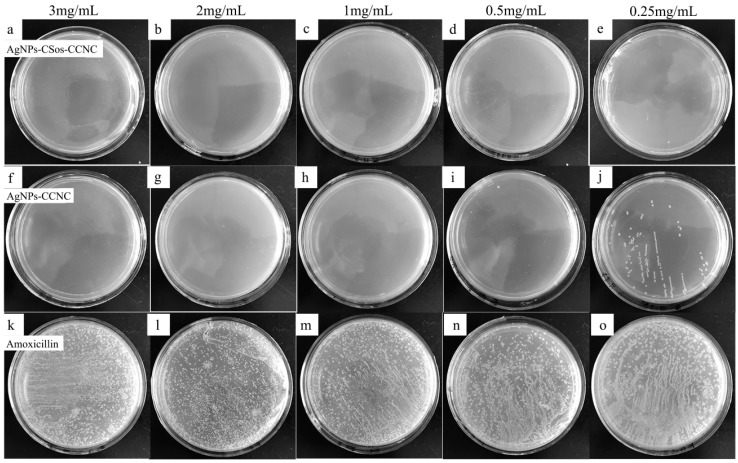
Antimicrobial activity of AgNPs-CSos-CCNC, AgNPs-CCNC and amoxicillin evaluated with *Klebsiella pneumoniae.* The antimicrobial agent concentrations were shown on the top of each plate. (**a**–**e**) the colony growth with AgNPs-CSos-CCNC, (**f**–**j**) the colony growth with AgNPs-CCNC, (**k**–**o**) the colony growth with amoxicillin.

The result of MIC test for the *Klebsiella pneumoniae* system is shown in Figure 8. The antimicrobial agent concentrations are shown on the top of each plate. The plates in the top row show the community growth with AgNPs-CSos-CCNC, while the plates in the middle row show the community growth under AgNPs-CCNC. The plates in the bottom row show the community growth under amoxicillin as positive control.

The bacterial community density decreased with increasing amounts of AgNPs. The *Klebsiella Pneumoniae* colonies disappeared when 0.5 mg/mL AgNPs-CSos-CCNC was used (Figure 8d), while 1 mg/mL of AgNPs-CCNC was acquired to reach the same effect (Figure 8h). The MIC for AgNPs-CSos-CCNC was between 0.25–0.5 mg/mL compared to 0.5–1 mg/mL for AgNPs-CCNC. The *Klebsiella pneumoniae* colonies were observed noticeably when the concentration of amoxicillin was 3 mg/mL. Therefore, the antimicrobial activity of AgNPs-CSos-CCNC was about two times better than that of AgNPs-CCNC.

The enhanced antibacterial properties of AgNP complexes should be attributed to the function of CSos achieved by enlarging the specific surface area of CCNC and changing the negative charge on the surface of the base material into positive. During the sedimentation process of AgNPs, part of Ag^+^ can be deposed onto the surface of CSos instead of CCNC, which provides larger interfacial adhesion surface, leading to the fact that AgNPs deposited on CSos-CCNC are smaller than the ones deposited on CCNC. Earlier studies have found that AgNPs with small diameters had a better effect on bactericidal [26,27]. Moreover, surface charge is a decisive factor in determining the antibacterial properties of AgNP complexes because positively charged AgNP complexes can easily trap bacterial cell wall and kill the bacterium [26], On the contrary, negatively charged CCNC can negatively influence the antibacterial performance of AgNPs by forming electrostatic repulsion during the process of trapping bacterium.

### 3.7. Cytotoxicity Evaluation

**Figure 9:**
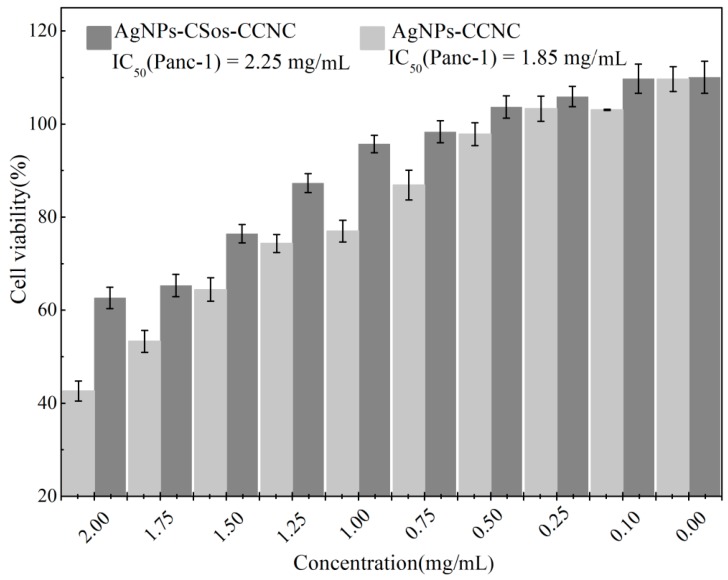
The cytotoxicity of the AgNPs-CSos-CCNC and the AgNPs-CCNC hybrid on Panc-1 cells using the CCK8 assay.

The preliminary cytotoxicity tests of the resultant nanomaterials were carried out using Panc-1 cells. Panc-1 cells were treated with the two AgNP complexes at ten different concentrations for 24 h. Cell viability was evaluated by CCK8 assay. Activities were given as IC_50_-values. In Figure 9, both AgNPs-CCNC and AgNPs-CSos-CCNC showed some toxicity to Panc-1 cell and the cytotoxicity was more obvious with increased quantity of nanoparticles. Additionally, AgNPs-CSos-CCNC was less toxic to Panc-1 cells compared to AgNPs-CCNC when the concentration increased up to 0.75 mg/mL. The IC_50_ (Panc-1) of AgNPs-CCNC and AgNPs-CSos-CCNC were 1.85 mg/mL and 2.25 mg/mL, respectively.

**Figure 10:**
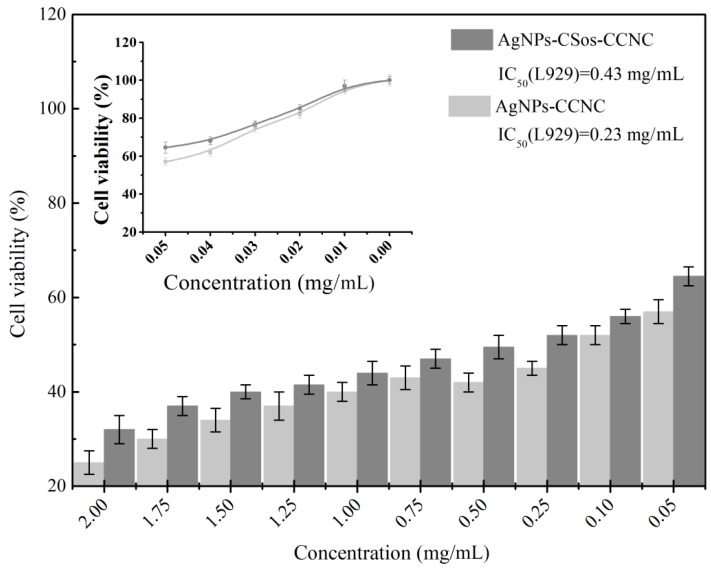
The cytotoxicity of the AgNPs-CSos-CCNC and the AgNPs-CCNC hybrid on L929 cells using the CCK8 assay.

To test the cytotoxicity of two types of AgNPs on the normal cells, mouse fibroblast cells (L929) were treated with different concentrations of the two AgNP complexes for 24 h, and cell viability was assessed by CCK-8 assay. Activities were given as IC_50_-values. As shown in Figure 10, both AgNPs-CCNC and AgNPs-CSos-CCNC showed certain toxicity to L929 cell. The cytotoxicity was more obvious with increased quantity of nanoparticles. The cell viability of L929 cells treated with 0.02 mg/mL of AgNPs-CCNC and AgNPs-CSos-CCNC was 82.36% and 85.11%, respectively. AgNPs-CCNC and AgNPs-CSos-CCNC at 0.05 mg/mL were obviously toxic to cells, with viability decreased to 56.84% and 64.53%. The IC_50_ (L929) of AgNPs-CCNC and AgNPs-CSos-CCNC were 0.43 mg/mL and 0.23 mg/mL, respectively. Two types of AgNPs were highly toxic to normal cells than cancer cells. The result indicated that coating with CSos caused the reduced cell toxicity of AgNPs, although further research is needed for a thorough explanation.

## 4. Conclusions

Two AgNP stabilizers—CCNC and CSos-CCNC—were successfully fabricated. The effects of the two stabilizers on the physico-chemical properties, antibacterial properties, and cytocompatibility of AgNPs were examined. FTIR investigation indicated that the oxidation of CCNC fabricated by one-step acid hydrolysis was 38%, while the 62% -OH residue contributed to the formation of CCNC bundles. Most of the hydroxyl groups reacted with chito-oligosaccharides. The average diameter of AgNPs stabilized by CCNC was approximately double that of AgNPs stabilized by CSos-CCNC, with the diameter distribution of the former clearly wider than that of the latter. This may be ascribed to the fact that CSos enlarged the surface area of CCNC. Through TGA curves, we concluded that the thermal stability of AgNPs-CSos-CCNC was superior to that of AgNPs-CCNC. Furthermore, it was found that the amounts of AgNPs in AgNPs-CSos-CCNC were less than 11 wt.%, whereas the counterpart amounts in AgNPs-CCNC were lower than 8 wt.%. AgNPs did not affect the crystal structure of CSos-CCNC and CCNC. Besides, the antibacterial activity of AgNPs-CSos-CCNC against *E. coli, Klebsiella pneumoniae*, and *S. aureus* was about 3, 2, and 3 times that of AgNPs-CCNC, respectively. The cytocompatibility of AgNPs-CSos-CCNC was better than that of AgNPs-CCNC, indicating that CSos-CCNC could lower the deposition of AgNPs more than CCNC. This research opens an avenue for developing a nanocomposite system to manufacture environmentally friendly and biocompatible stabilizers to achieve high antibacterial performance and lower deposition of AgNPs.

## Figures and Tables

**Table 1:** Thermal properties results of silver nanoparticles-carboxy-cellulose nanocrystals (AgNPs-CCNC) and silver nanoparticles-chito-oligosaccharides-carboxy-cellulose nanocrystals (AgNPs-CSos-CCNC).

Code	T_onset_ (°C)	T_max_ (°C)
AgNPs-CCNC	212	330.18
AgNPs-CSos-CCNC	199	353.41
